# Supplementary material for: 14-3-3 Activated Bacterial Exotoxins AexT and ExoT Share Actin and the SH2 Domains of CRK Proteins as Targets for ADP-Ribosylation
Source: Pathogens. 2022 Dec 8;11(12):1497. doi: 10.3390/pathogens11121497 (PMC9787417; doi:10.3390/pathogens11121497)
Supplement: Supplementary file 1 [file pathogens-11-01497-s001.zip › pathogens-2057737-supplementary.pdf]

## Supplementary Information

accompanying

# 14-3-3 Activated Bacterial Exotoxins AexT and ExoT Share Actin and the SH2 Domains of CRK Proteins as Targets for ADP-Ribosylation

**Carmen Ebenwaldner, Peter Hornyak, Antonio Ginés García Saura, Archimede Torretta and Herwig Schüler**

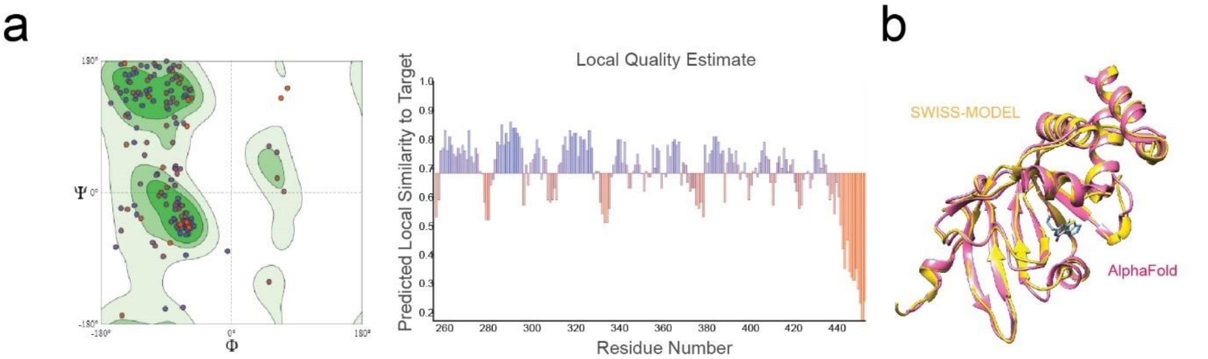


**Figure S1 (pertaining to Figure 1 of the main text):** Quality of the AexT-ART homology model constructed with SWISS-MODEL. (**a**) Ramachandran plot and per-residue quality estimate with global quality score (QMEANDisCo) of 0.68 represented as the average of the local quality estimate. (**b**) Superimposition with the AlphaFold model. RMSD across all 198 Cα atom pairs: 1.403Å (0.772Å between 182 pruned atom pairs).

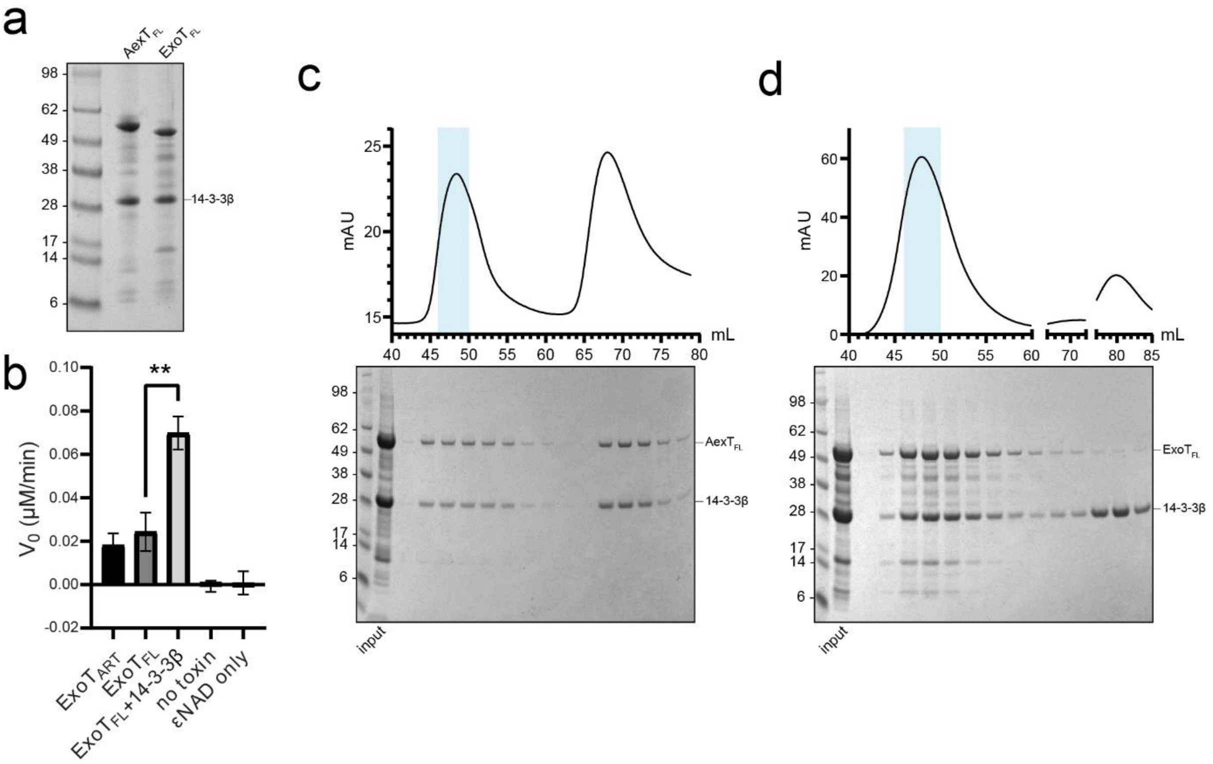


**Figure S2 (pertaining to Figure 2 of the main text):** Steps in the recovery of full-length toxins from *E.coli* inclusion bodies. (**a**) Coomassie stained SDS-PAGE gel showing the purity of the refolded exotoxins before size exclusion chromatography. (**b**) The addition of 14-3-3β during the refolding process significantly improves the activity of refolded ExoT. Processing rates were determined in assays containing 200 nM toxin, 500 nM 14-3-3β, 5 µM CRK SH2 domain, and 40 µM εNAD+. **, adjusted P value = 0.002, determined with ordinary one-way ANOVA. (**c, d**) Typical SEC profiles of (c) AexT and (d) ExoT in complex with 14-3-3β. A substantial amount of protein elutes in the void volume of the column (around 48 ml, indicated in blue; column: Hiload 16/600 Superdex 200 pg). The complex of interest elutes around 70 ml, followed by excess 14-3-3β.

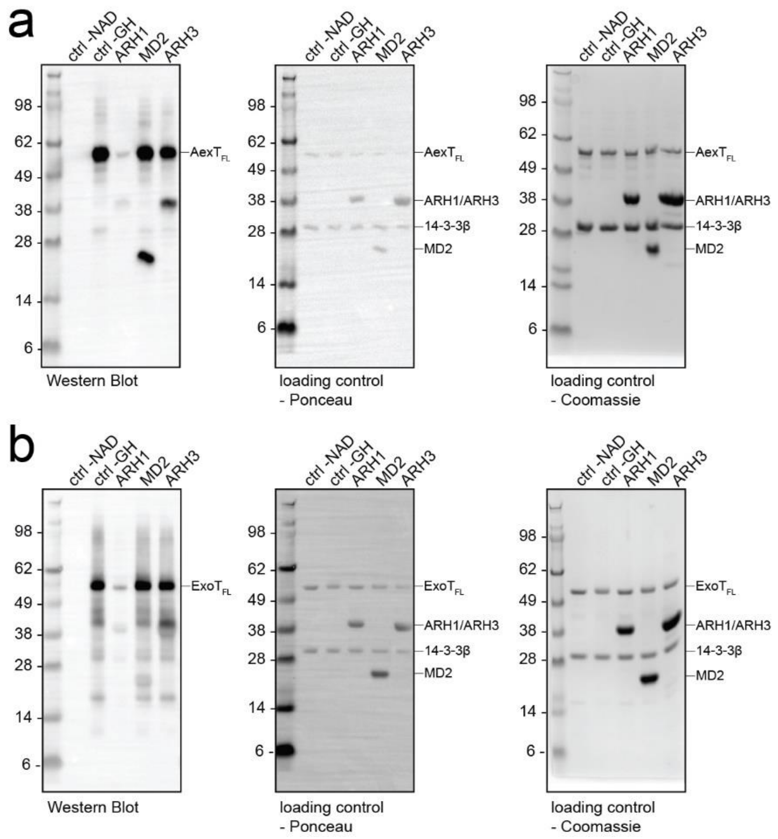


**Figure S3 (pertaining to Figure 4 a,b of the main text):**. Uncropped Western blot images and loading controls for Figure 4a,b. (a) AexT; (b) ExoT.

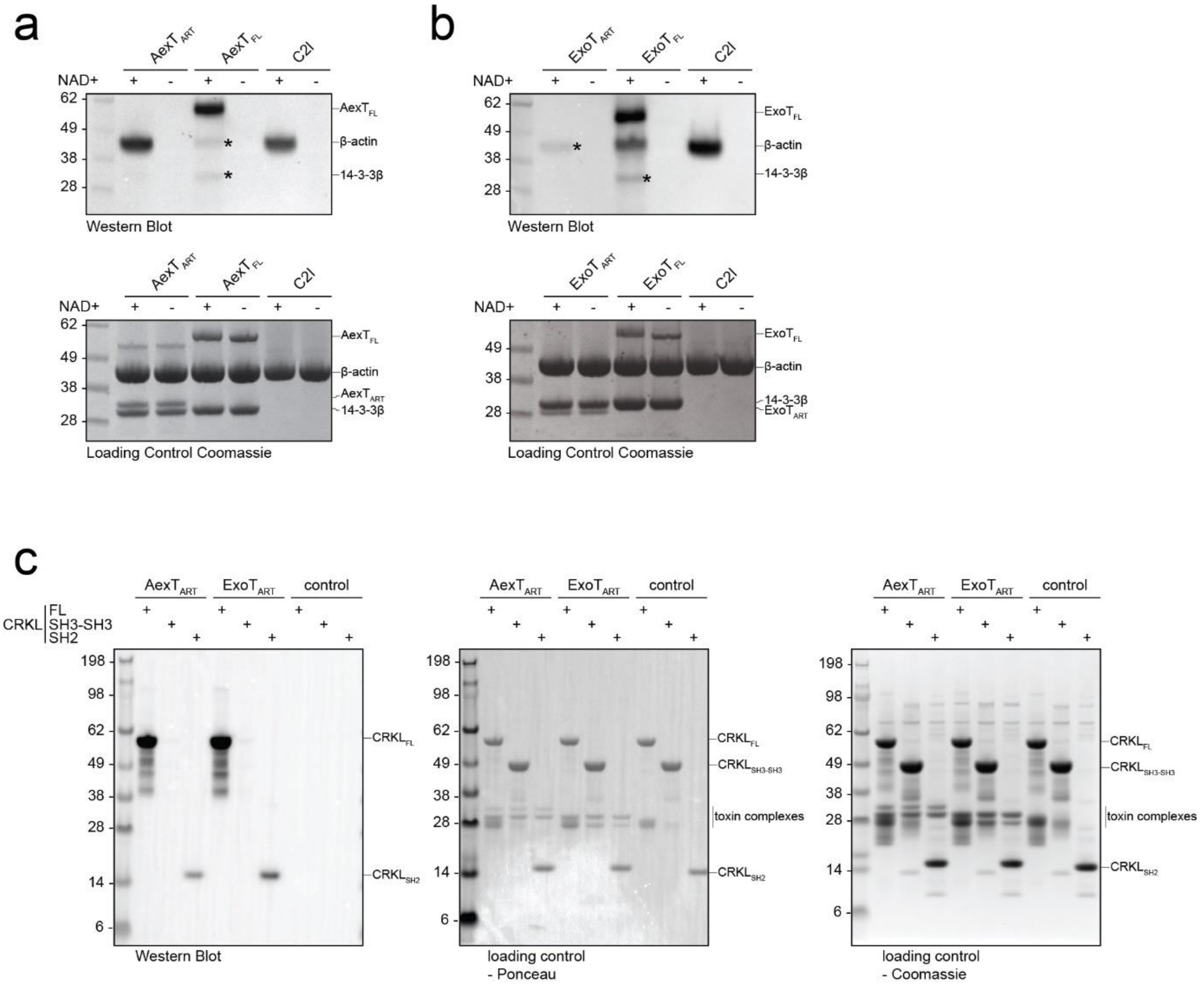


**Figure S4 (pertaining to Figure 5 of the main text):** Uncropped Western blot images and loading controls for Figure 5 a,b,c.

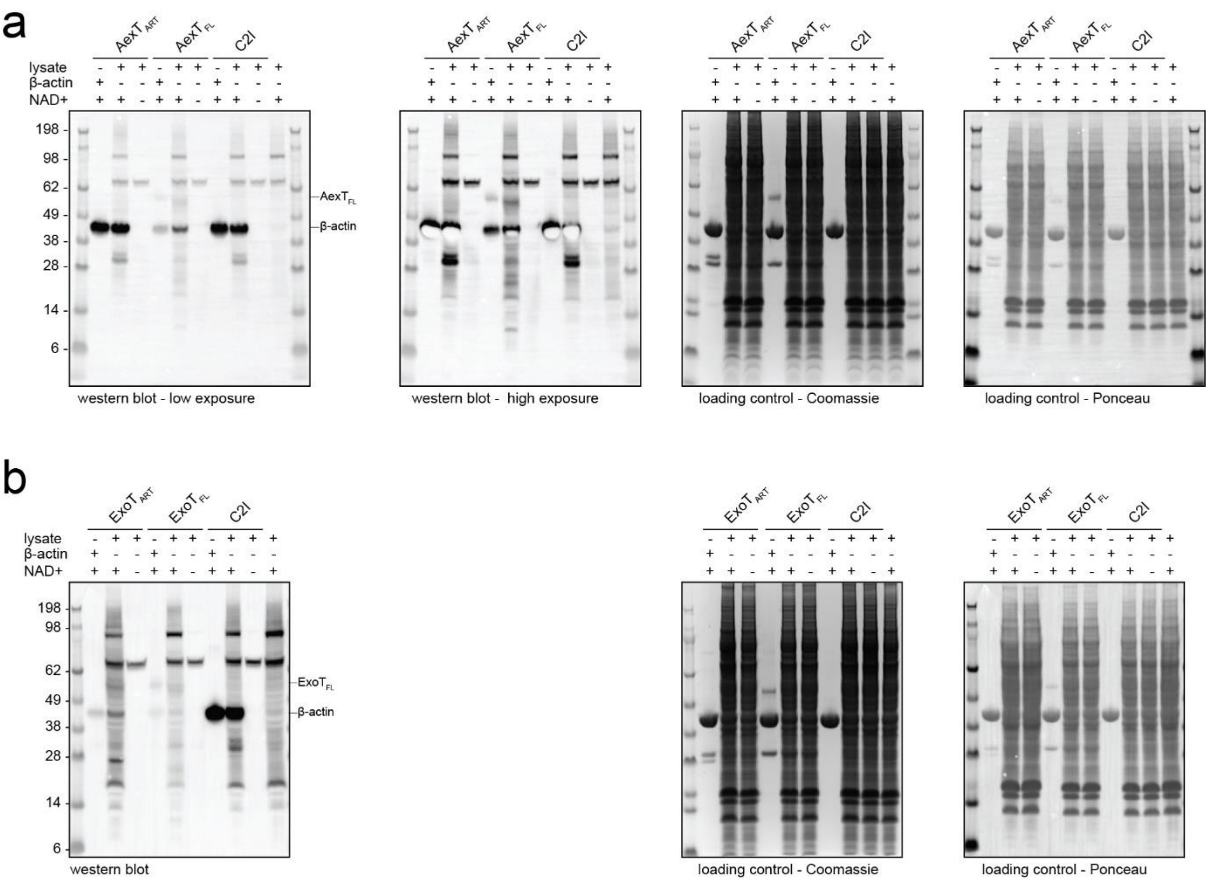


**Figure S5 (pertaining to Figure 6 of the main text):** Uncropped Western blot images, alternative exposures and loading controls for Figure 6 a,b.
